# Improving Prescription Completeness and Reducing Prescription Errors in a Neonatal Intensive Care Unit: A Quality Improvement Initiative From a Tertiary Care Centre in North India

**DOI:** 10.7759/cureus.92039

**Published:** 2025-09-11

**Authors:** Sanchita Dhawan, Shantanu Shubham, Naini Puri, Syed Moiz Ahmed, Divya Mishra, Aayushi Joshi, Richa Joshi, Girish Gupta

**Affiliations:** 1 Pediatrics, Graphic Era (Deemed to be University), Dehradun, IND; 2 Neonatology, Graphic Era (Deemed to be University), Dehradun, IND; 3 Obstetrics and Gynaecology, Graphic Era Deemed to be University, Dehradun, IND

**Keywords:** medication safety, neonatal intensive care unit, neonatology, prescription completeness, prescription errors, quality improvement

## Abstract

Objective: Incomplete prescriptions are a frequent contributor to medication errors in neonatal intensive care units (NICUs), where patient vulnerability and complex dosing requirements increase the risk. An initial assessment at our unit identified a gap in prescription completeness, leading to the development of a structured quality improvement (QI) project.

Methods: Using the Point of Care Quality Improvement (POCQI) and Institute for Healthcare Improvement (IHI) Model for Improvement, we implemented a 24-week QI study divided into baseline (four weeks), intervention (16 weeks), and sustenance (four weeks) phases. A 10-point prescription checklist was developed, incorporating NICU-specific parameters such as oxygen targets, nutraceuticals, and developmentally supportive care (DSC). Root cause analysis was performed using fishbone and Pareto charts. Interventions were tested through four sequential plan-do-study-act (PDSA) cycles and included training sessions, pre-printed templates, DSC integration, and structured prescribing of supplements. Daily audits were conducted on randomly selected prescriptions, with correctness scored and tracked using run and control charts.

Results: A total of 672 prescriptions were analyzed. The baseline mean score of prescription completeness was 4.54/10, with only 11.6% achieving ≥70% completeness. Scores improved progressively across PDSA cycles, reaching a sustained mean of 8.4/10 in the final phase. The proportion of prescriptions scoring ≥70% rose to 100%, with a score of 10/10 in a few prescriptions during sustenance. No significant increase in prescription completion time was noted.

Conclusion: Structured, low-cost interventions significantly improved prescription completeness in a resource-constrained NICU. Institutionalizing training, checklist use, and regular audits may help sustain these improvements.

## Introduction

Background

The administration of medications forms a vital and inseparable part of patient management. This process gains even greater significance within intensive care units, where a substantial number of medications are crucial for sustaining life. Medication delivery follows a structured sequence of steps, including prescribing, transcribing, dispensing, administering, and subsequent monitoring [1]. The Institute of Medicine estimates that medical errors contribute to approximately 44,000 to 98,000 deaths each year in the United States, with prescription-related issues comprising a significant proportion of these events [2]. In India, the prevalence of prescription inaccuracies is notably high. Jain et al. (2004) reported an error rate of 9.6 per 100 prescriptions in a tertiary care hospital, with even higher rates observed in neonatal emergency departments [3]. Similarly, an audit conducted in a neonatal care unit in Kolkata revealed a baseline prescription inaccuracy rate of 71.1 per 100 prescriptions [4]. The inherent vulnerability and developmental immaturity of neonatal organ systems further amplify the risk of adverse outcomes in this already precarious environment [5]. Prescription writing marks the initial phase of the medication administration process. However, this critical step can also serve as the origin point for subsequent drug administration errors. A prospective study conducted in a major tertiary neonatal intensive care unit (NICU) in Scotland revealed that poor prescription practices accounted for 71% of all medication errors [6]. Other studies have reported a wide variation in prescription error rates, ranging from 14% to 74% [7-11].

Available knowledge

Prescription writing in neonatal practice is complicated by the considerable variability in recommended drug dosages. For many medications commonly used in neonates, dosage guidelines differ significantly across standard reference sources and often vary based on the infant’s gestational and postnatal age. A recent review analyzing strategies to minimize medication errors highlighted that most interventions focused primarily on reducing prescription errors as a key approach to lowering the overall incidence of medication-related mistakes [12]. Several system-level factors contribute to prescription errors, including high clinical workload, non-standardized prescription formats, inconsistent prescriber training, and the lack of routine audit and feedback mechanisms [4]. Structured quality improvement (QI) initiatives have demonstrated significant success in addressing these issues. Interventions such as implementing standardized prescription templates, conducting regular training sessions, and establishing feedback loops have been associated with substantial improvements in prescription accuracy [13]. Global guidelines emphasize the importance of safe prescribing practices, advocating for continuous professional education, use of validated prescription checklists, and integration of electronic prescribing systems wherever feasible [14].

Study rationale

Studies have documented that medication errors, including incorrect dosages, incomplete prescriptions, and omissions, are frequent in neonatal settings and contribute to adverse clinical outcomes [6,15]. Errors range from missing patient identifiers and drug details to improper documentation of dilution volumes and infusion rates, often resulting in medication-related adverse events and extended hospital stays.

A retrospective assessment was conducted in our NICU at Graphic Era Institute of Medical Sciences (GEIMS), Dehradun, Uttarakhand, India, to evaluate the completeness of prescriptions using a structured, comprehensive checklist (see Appendix A). The checklist encompassed essential prescription components, including standard elements (e.g., date and time, patient identifiers, medication details, and dosing schedule) as well as NICU-specific aspects such as documentation of oxygen and SpO₂ targets, prescription of nutraceuticals, and developmentally supportive care (DSC) measures like kangaroo mother care (KMC) and infant positioning assessments. Upon review of 50 prescriptions over 15 days, the mean completeness score per prescription was approximately 5 out of 10, indicating that only 50% of critical parameters were adequately addressed.

Recognizing the implications, a QI project was initiated to enhance prescription completeness. Drawing from evidence that interventions targeting prescription errors can significantly reduce overall medication errors [12], this project aims to implement structured interventions in prescription practices within our NICU. The specific, measurable, achievable, relevant, and time-bound (SMART) aim of this study was to increase the prescription correctness rate in the NICU of GEIMS from 50% to 70% over a period of 24 weeks.

## Materials and methods

Study setting

This study was conducted over a period of six months after obtaining ethical clearance from the Institutional Review Board of GEIMS (approval no. GEIMS/IRB/RP/24/2025). All neonates admitted to the NICU during the study period were included in the study, while those admitted to the postnatal wards were excluded from the analysis.

**Figure 1:**
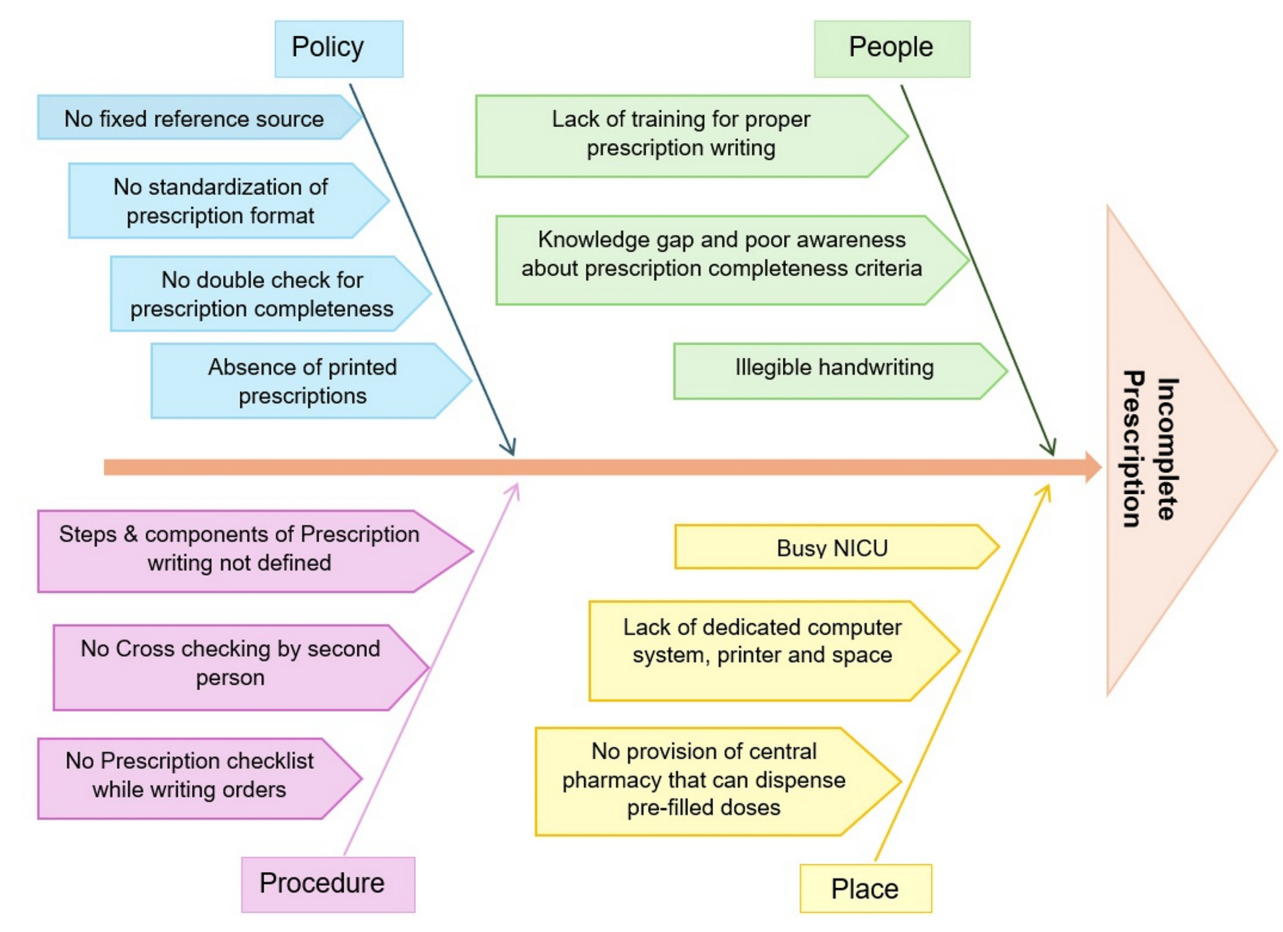
Fishbone (Ishikawa) diagram on the root cause analysis of incomplete prescriptions in NICU

The NICU at our tertiary care teaching hospital is a 25-bed level III facility that manages approximately 1,500 deliveries annually. The unit provides continuous, high-dependency care to critically ill neonates (both inborn and outborn) and is staffed by neonatology consultants, fellows, pediatric residents, and trained neonatal nursing personnel. Despite the importance of correct prescriptions, multiple operational and contextual challenges have contributed to inconsistencies in prescription practices. These include a consistently high patient volume, frequent turnover, and rotation of medical staff with varying levels of experience, and the challenges associated with the evolving infrastructure of a newly established NICU. To systematically explore the underlying causes of incomplete prescriptions, a root cause analysis was undertaken using a fishbone (Ishikawa) diagram (Figure 1).

**Figure 2:**
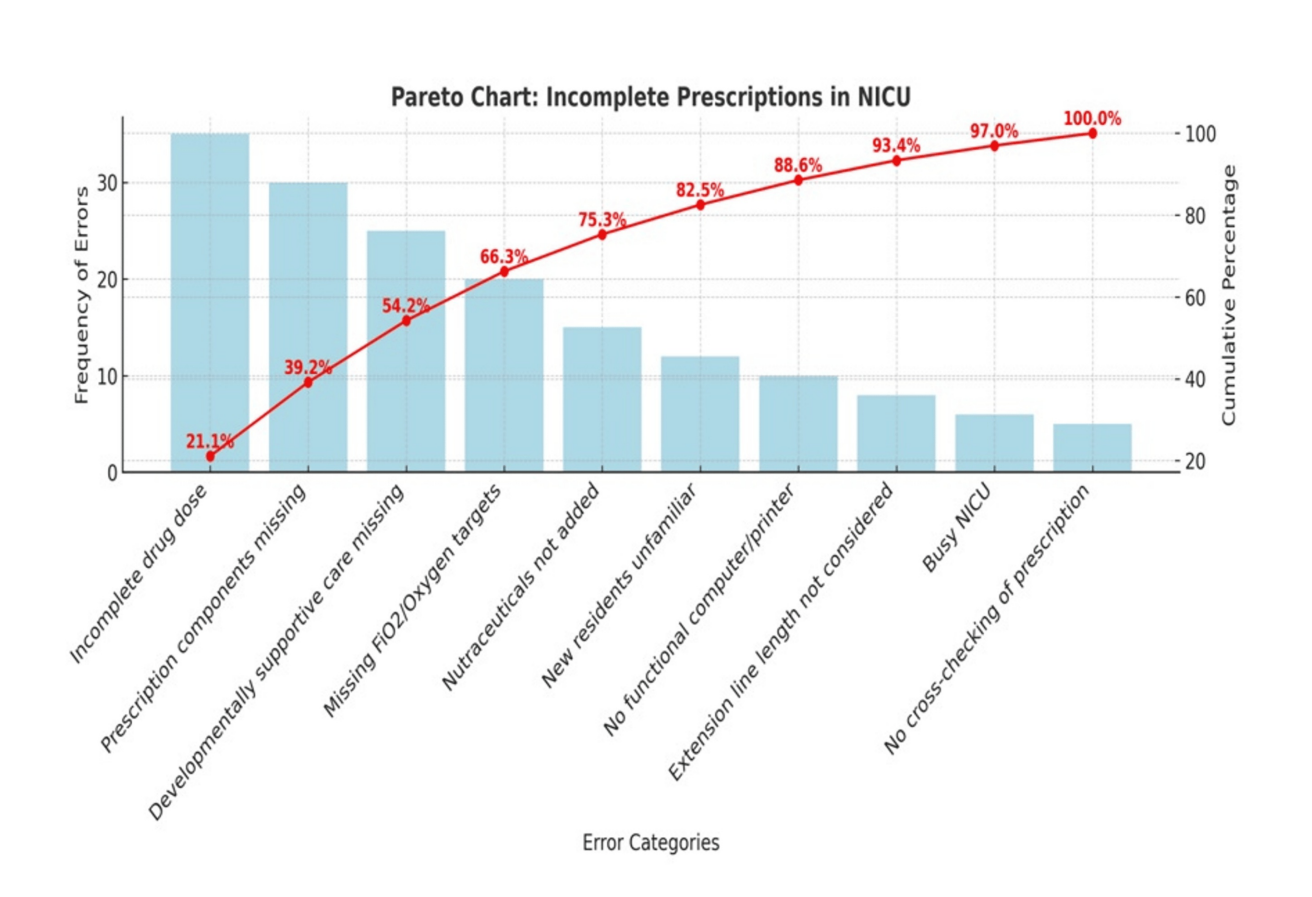
Pareto chart on the frequency of error categories in incomplete prescriptions

The identified contributing factors were categorized under four domains: people, policy, procedure, and place. To complement the qualitative findings of the fishbone analysis, a Pareto chart was developed to quantitatively assess the frequency of different types of prescription errors (Figure 2). The chart revealed that four major error categories, i.e., incomplete drug dosing, missing prescription components, absent fraction of inspired oxygen (FiO₂)/oxygen targets, and lack of DSC documentation, accounted for over 66% of total errors. This finding, consistent with the Pareto principle, emphasized that most of the problems were attributable to a small subset of root causes, thereby allowing the team to prioritize these high-yield areas for intervention. Informed by these findings, a QI initiative was launched, employing multiple plan-do-study-act (PDSA) cycles to address and iteratively improve prescription completeness and correctness.

Intervention

This QI initiative followed the Point-of-Care Quality Improvement (POCQI) framework in conjunction with the Institute for Healthcare Improvement (IHI) Model for Improvement [16,17]. The study was implemented in four structured steps: (1) identifying the problem and forming a team, (2) analyzing and measuring the quality of care, (3) developing and testing changes, and (4) sustaining improvements. The total study duration was 24 weeks, divided into three distinct phases: baseline (four weeks), intervention (16 weeks), and sustenance (four weeks). The extended intervention phase facilitated the implementation of four iterative PDSA cycles.

A multidisciplinary QI team was established, consisting of two neonatology consultants, two fellows, and two nursing officers. The team developed a structured 10-point checklist to assess prescription correctness, with each parameter representing a critical component of neonatal prescriptions (see Appendix A). Each correctly documented parameter was awarded 1 point, while incorrect or missing entries scored zero. To ensure objective evaluation, four prescriptions were randomly selected each day using a Microsoft Excel (Microsoft Corp., Redmond, WA, USA) random number function. Prescription audits were conducted independently by the hospital’s quality team during morning clinical rounds, which was different from the QI team involved in the development of the prescription checklist. The assessors were not involved in patient care, ensuring blinding from the treating team and minimizing observer bias. In total, 672 prescriptions were evaluated across all three phases: 112 in the baseline phase, 448 during the intervention phase, and 112 in the sustenance phase.

PDSA Cycle One

**Figure 3:**
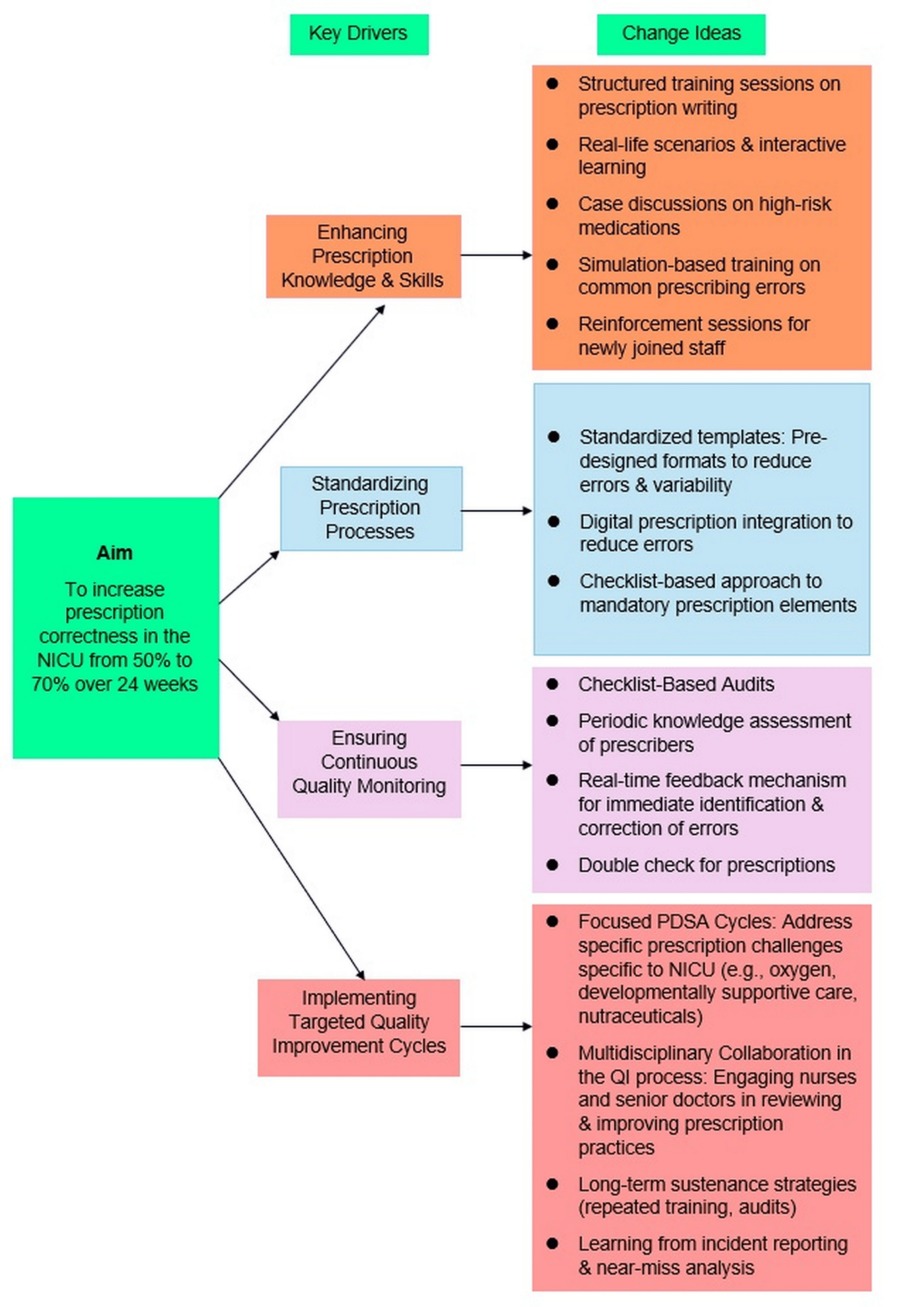
Driver diagram on the key drivers and change ideas for improving prescription correctness

The first PDSA cycle focused on improving basic prescription writing practices by enhancing the knowledge and skills of NICU healthcare providers. A driver diagram was prepared to systematically map the factors influencing prescription correctness and to guide targeted interventions for improvement (Figure 3). Weekly interactive teaching sessions were organized for residents and nurses, emphasizing the essential components of a complete prescription, such as patient identifiers, date and time, medication name, dosage, frequency, route of administration, and the prescriber’s signature. These sessions incorporated real-life NICU scenarios, hands-on exercises, and constructive feedback to build confidence and reinforce correct practices. Common pitfalls such as illegible handwriting, missing details, and incorrect dosages were discussed, along with practical strategies for avoidance. To ensure consistency in dosing, the Micromedex® Neofax® manual was introduced as the standard reference source for all prescribers. This cycle laid the foundation for a shared understanding of prescription completeness, addressing the knowledge gaps and variability observed during the baseline phase.

PDSA Cycle Two

The second PDSA cycle targeted prescription variability by implementing a structured, pre-printed prescription template for commonly used NICU medications (see Appendix B). It was stored digitally on the computer system used for prescription entry to facilitate access and ease of use. To promote consistent adoption, the intervention was reinforced through weekly meetings and visual reminders displayed near the prescription entry points. This cycle significantly improved standardization and minimized errors arising from inconsistent formatting, thus addressing procedural gaps and documentation-related issues highlighted in the root cause analysis.

PDSA Cycle Three

In the third PDSA cycle, efforts were directed toward improving the prescription of oxygen therapy and integrating DSC into routine neonatal care planning. Weekly training sessions were conducted on evidence-based oxygen therapy, focusing on defining appropriate saturation of peripheral oxygen (SpO₂) target ranges, outlining weaning protocols, and implementing strategies to prevent complications such as retinopathy of prematurity (ROP). Parallelly, emphasis was placed on the inclusion of DSC elements, such as noise control, KMC, and optimal positioning, in both clinical practice and written prescriptions. This cycle led to notable improvements in the completeness of oxygen therapy documentation and promoted a more holistic approach to neonatal care.

PDSA Cycle Four

The fourth PDSA cycle addressed the frequent omission and inaccuracy of nutraceutical prescriptions, particularly involving essential neonatal supplements like vitamins, minerals, and trace elements. Educational sessions were conducted weekly, focusing on the evidence-based prescribing of key supplements tailored to gestational and postnatal age. The intervention resulted in improved accuracy and consistency of nutraceutical entries in prescriptions, aligning with best practices for comprehensive neonatal nutritional support.

Measures

The objective scoring of prescription correctness was taken as the process measure of our study. This was assessed using a structured 10-component checklist, with each correctly documented parameter receiving a score of 1 and incorrect or missing parameters receiving a score of 0, resulting in a maximum possible score of 10 per prescription. The mean prescription correctness score was calculated daily based on the mean of four randomly selected prescriptions. The outcome measure was defined as the percentage of prescriptions achieving ≥70% completeness, corresponding to a score of 7 or more out of 10. To ensure that the interventions did not inadvertently increase workload or disrupt clinical care, the balancing measure was the average time taken to complete a prescription, which was periodically assessed to monitor for any negative impact on workflow efficiency.

Data acquisition and analysis

Four prescriptions were randomly selected and scored daily using the structured checklist. The mean value of the four prescriptions was plotted both on a run chart and a statistical process control (SPC) chart daily to visualize trends and variations in prescription correctness over time. Control limits for the SPC charts were recalculated at four-week intervals to reflect changes during each phase of the study. Data collection was carried out using a structured case record form, and entries were made in Microsoft Excel version 2016 (Microsoft Corp., Redmond, WA, USA). Data analysis was performed using STATA version 14 (StataCorp, College Station, TX, USA). For statistical charting, SPC charts were generated using QI Macros software (KnowWare International, Denver, CO, USA), while run charts were created using a publicly available Excel template from the IHI website (http://www.ihi.org/resources/pages/tools/runchart.aspx).

## Results

**Figure 4:**
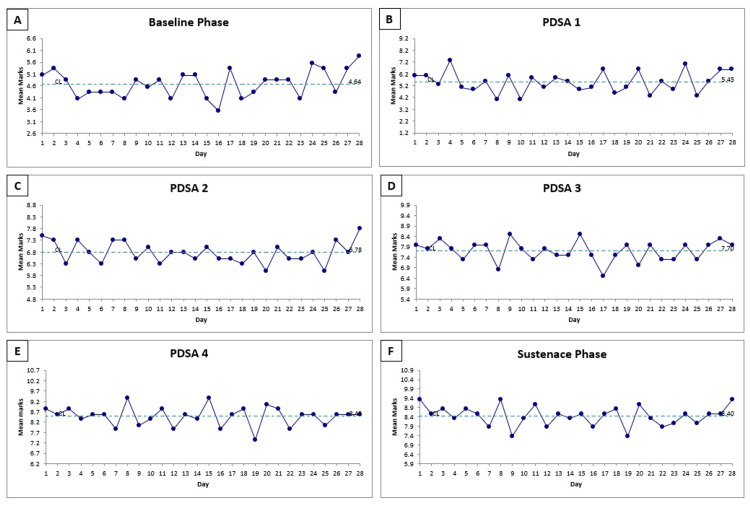
Run charts on daily mean prescription scores across study phases Panels A to F represent daily mean prescription scores during each phase. Progressive improvement is observed across phases, with consistent scores maintained during the sustenance phase. PDSA: Plan-do-study-act

**Figure 5:**
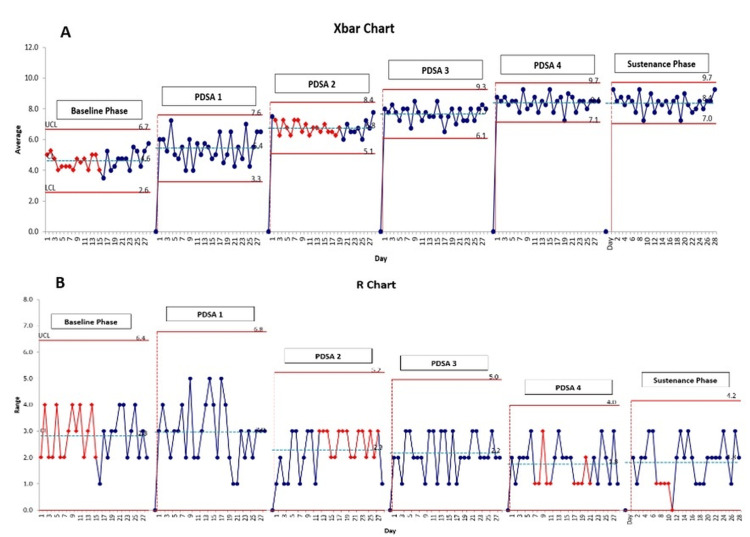
Control charts on monitoring prescription correctness and variation over time A: The X-bar chart displays the average prescription score with recalculated control limits across phases; B: The R chart shows decreasing variability in scores, indicating improved process consistency through PDSA cycles. PDSA: Plan-do-study-act

A total of 672 prescriptions were evaluated over the 24-week study period, 112 each during the baseline and sustenance phases, and 448 during the 16-week intervention phase, which was divided into four sequential PDSA cycles. The prescription correctness scores were plotted daily using run charts for each phase (Figure 4, panels A to F), while overall performance trends and variability were assessed using SPC charts, including X-bar and R charts (Figure 5, panels A to B).

During the baseline phase, the mean prescription correctness score was 4.54 out of 10, with an upper control limit (UCL) of 6.7 and a lower control limit (LCL) of 2.6, indicating substantial variability in prescription quality. Following the implementation of PDSA cycle one, which focused on structured training sessions for prescription writing, the mean score increased to 5.45, accompanied by recalculated control limits (UCL: 7.6, LCL: 3.1), reflecting an early positive shift. In PDSA cycle two, the introduction of standardized printed templates further enhanced prescription consistency, raising the mean score to 6.78 (UCL: 8.4, LCL: 5.1). Continued improvements in PDSA cycle three, targeting oxygen prescription accuracy and integration of DSC, elevated the mean score to 7.30 (UCL: 9.2, LCL: 6.1). Improvement was observed in PDSA cycle four, which addressed documentation of nutraceuticals, achieving a mean score of 8.40 with narrowed control limits (UCL: 9.7, LCL: 7.1). This enhanced performance was successfully sustained during the sustenance phase, where the mean score remained stable at 8.40 (UCL: 9.7, LCL: 7.0), signifying a durable shift toward consistently complete and high-quality prescriptions.

The X-bar chart (Figure 2, panel A) demonstrated a progressive upward shift in the mean prescription correctness scores across the intervention period. Although no individual data points crossed the UCL, special cause variation was observed as per standard SPC rules, evident through consecutive data points consistently lying above the baseline mean and highlighted as red points on the chart. This pattern indicated a statistically significant improvement attributable to the implemented interventions, rather than random variation. Furthermore, the R chart (Figure 2, panel B) displayed a noticeable narrowing of the range over successive PDSA cycles, suggesting reduced variability and increased consistency in prescribing practices.

The outcome measure was the percentage of prescriptions achieving ≥70% correctness, defined as a score of 7 or more out of 10. In the baseline phase, only 13 prescriptions (11.6%) met the ≥7 threshold, and none achieved a perfect score of 10. Following the implementation of PDSA cycle one, this improved to 31 prescriptions (27.7%), still with no perfect scores. In PDSA cycle two, after introducing standardized printed templates, 66 prescriptions (58.9%) reached the desired score range. PDSA cycle three, which focused on oxygen prescription and developmentally supportive care, yielded further gains with 92 prescriptions (82.1%) scoring ≥7, though no prescriptions achieved a perfect 10 in either PDSA cycle two or PDSA cycle three. Improvement was seen in PDSA cycle four, which addressed nutraceutical prescription accuracy. Here, all 112 prescriptions (100%) met the ≥7 criteria, and 11 prescriptions achieved a perfect score of 10/10. This improvement was successfully sustained post-intervention, with the sustenance phase also achieving 100% completeness (112/112 prescriptions scoring ≥7), and 13 prescriptions reaching the perfect score benchmark.

The balancing measure for the study was the average time taken to complete a prescription, assessed informally during each PDSA cycle. Clinical feedback indicated that although the introduction of printed templates, structured training, and checklists added minor documentation steps, they did not cause any significant delay or disruption in clinical workflow.

## Discussion

Neonates are particularly susceptible to medication errors due to their physiological immaturity, the necessity for weight-based and gestational-age-based dosing, and the frequent use of unlicensed or off-label medications, often requiring complex dilutions and manipulations [4,18]. Our QI initiative, structured using the POCQI and IHI models, sought to address prescription completeness in a resource-constrained level III NICU through simple, low-cost, replicable interventions.

The baseline data from our unit, where only 13 of 112 prescriptions achieved a correctness score of ≥7 out of 10, highlights the magnitude of the problem. This aligns with the findings of Eslami et al. [7], who reported that over 70% of neonates experienced at least one medication error, with incorrect dosages and missing time/date details being the most frequent. In our study, structured training (PDSA cycle one) and the introduction of a printed template (PDSA cycle two) led to steady improvements in prescription correctness. The effectiveness of structured prescription formats in improving accuracy has also been supported by Pallás et al., who demonstrated significant reductions in errors following the implementation of standardized preprinted order forms in neonatal units [8]. Further gains were seen in PDSA cycle three and PDSA cycle four, where we targeted oxygen prescription accuracy and the integration of nutraceutical components. These targeted interventions resonate with previous studies, which emphasize that clarity in dosage, administration details, and standardization significantly reduce error rates [8,19]. Additionally, our improvement trajectory mirrors the results of Mondal et al. [4] from a tertiary care center in Kolkata, who also reported a substantial reduction in medication errors through PDSA cycles and low-tech interventions. The sustenance phase maintained the prescription correctness, affirming the durability of system-level changes. Such sustainability reflects the value of participatory QI models and aligns with the review by Nguyen et al. [12], which concluded that no single intervention is superior but that multifactorial strategies consistently achieve a 50% to 70% error reduction across studies.

Notably, our project also observed a progressive increase in the number of prescriptions achieving a perfect score of 10/10, rising from zero in baseline and early PDSA cycles to 11 in PDSA 4 and 13 in the sustenance phase. While interventions such as clinical decision support systems and electronic prescribing platforms have demonstrated significant error reductions in high-income settings [10], our findings support that context-appropriate, manual interventions can be equally transformative in low-resource NICUs. The integration of structured training, user-friendly prescription templates, and inclusion of oxygen and nutritional components served as high-impact, low-cost solutions tailored to our local challenges.

This study’s strengths include its structured use of the POCQI model, multidisciplinary team involvement, daily prospective scoring, and progressive PDSA-driven interventions tailored to a resource-limited NICU. The use of a simple, reproducible checklist and sustained improvement over time highlight its practical applicability. A key strength of this study is its unique inclusion of NICU-specific components, such as oxygen targets, DSC, and nutraceuticals, into the prescription checklist, making it tailored to neonatal care needs.

This study has several limitations. First, it was conducted in a single-center NICU, which may limit the generalizability of findings to other settings with different resources, patient profiles, or prescribing practices. Second, the absence of a control group restricts the ability to attribute improvements solely to the interventions implemented. Third, although a sustenance phase was included, the follow-up duration was only four weeks, which may not be sufficient to fully assess the long-term sustainability of improvements. Fourth, while prescription completeness was significantly enhanced, clinical outcome data, such as reduction in adverse drug events or measurable patient benefits, were not captured, limiting the direct correlation between improved documentation and improved patient safety. Limitations also include the challenges of scheduling ongoing training sessions and ensuring continuous monitoring and evaluation, which may affect the sustainability of the interventions. Finally, the interventions were context-specific, and while they proved effective in our NICU, their broader applicability across diverse healthcare settings requires further validation. 

## Conclusions

This quality improvement initiative significantly enhanced prescription completeness in a resource-limited NICU through structured, low-cost, and replicable interventions. To sustain the positive gains achieved, periodic training sessions, continued use of the prescription checklist, and regular audit-feedback mechanisms should be incorporated as part of routine NICU practice. These ongoing measures will help maintain high standards of prescription accuracy and patient safety. Additionally, fostering a culture of continuous quality improvement among staff is essential for long-term success in ensuring prescription completeness.

## Figures and Tables

**Figure 6:**
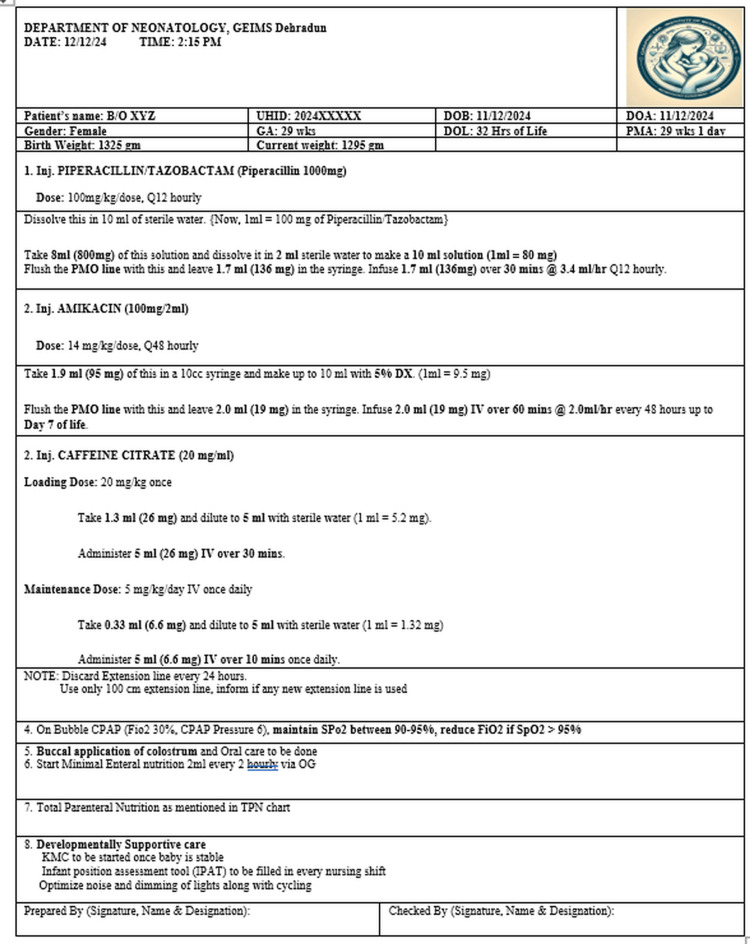
Sample of a structured prescription template GEIMS: Graphic Era Institute of Medical Sciences, FiO₂: Fraction of inspired oxygen, SpO_2_: Saturation of peripheral oxygen

## Appendices

Appendix A

Table 1 features the structured, comprehensive checklist used in this retrospective assessment. Each parameter represents a critical component of neonatal prescriptions. 

**Table 1:** Prescription checklist SpO_2_: Saturation of peripheral oxygen, DSC: Developmentally supportive care, KMC: Kangaroo mother care

S. No.	Components of a prescription	Correct	Incorrect
1.	Date and time		
2.	Name of baby, gestational and postnatal age		
3.	Name, dose, and route of drug		
4.	Diluent and its volume		
5.	Duration and rate of infusion		
6.	Frequency of medication administration		
7.	Inclusion of oxygen and SpO_2_ targets in the prescription		
8.	Nutraceuticals		
9.	DSC measure of KMC inclusion of infant positioning assessment tool (IPAT) in each nursing shift		
10.	Signature of resident and counter signature of consultant in-charge		

Appendix B

Figure 6 features the structured, pre-printed prescription template commonly used for NICU medications.
